# Capillary Electrophoresis-Based Functional Genomics Screening to Discover Novel Archaeal DNA Modifying Enzymes

**DOI:** 10.1128/AEM.02137-21

**Published:** 2022-01-25

**Authors:** Kelly M. Zatopek, Samantha L. Fossa, Katharina Bilotti, Paul J. Caffrey, Léa Chuzel, Alexandra M. Gehring, Gregory J. S. Lohman, Christopher H. Taron, Andrew F. Gardner

**Affiliations:** a New England Biolabs, Inc., Ipswich, Massachusetts, USA; Kyoto University

**Keywords:** *Archaea*, DNA modifying enzymes, DNA repair, functional genomics, enzyme discovery

## Abstract

It has been predicted that 30 to 80% of archaeal genomes remain annotated as hypothetical proteins with no assigned gene function. Further, many archaeal organisms are difficult to grow or are unculturable. To overcome these technical and experimental hurdles, we developed a high-throughput functional genomics screen that utilizes capillary electrophoresis (CE) to identify nucleic acid modifying enzymes based on activity rather than sequence homology. Here, we describe a functional genomics screening workflow to find DNA modifying enzyme activities encoded by the hyperthermophile Thermococcus kodakarensis (T. kodakarensis). Large DNA insert fosmid libraries representing an ∼5-fold average coverage of the T. kodakarensis genome were prepared in Escherichia coli. RNA-seq showed a high fraction (84%) of T. kodakarensis genes were transcribed in E. coli despite differences in promoter structure and translational machinery. Our high-throughput screening workflow used fluorescently labeled DNA substrates directly in heat-treated lysates of fosmid clones with capillary electrophoresis detection of reaction products. Using this method, we identified both a new DNA endonuclease activity for a previously described RNA endonuclease (Nob1) and a novel AP lyase DNA repair enzyme family (termed 'TK0353') that is found only in a small subset of Thermococcales. The screening methodology described provides a fast and efficient way to explore the T. kodakarensis genome for a variety of nucleic acid modifying activities and may have implications for similar exploration of enzymes and pathways that underlie core cellular processes in other Archaea.

**IMPORTANCE** This study provides a rapid, simple, high-throughput method to discover novel archaeal nucleic acid modifying enzymes by utilizing a fosmid genomic library, next-generation sequencing, and capillary electrophoresis. The method described here provides the details necessary to create 384-well fosmid library plates from Thermococcus kodakarensis genomic DNA, sequence 384-well fosmids plates using Illumina next-generation sequencing, and perform high-throughput functional read-out assays using capillary electrophoresis to identify a variety of nucleic acid modifying activities, including DNA cleavage and ligation. We used this approach to identify a new DNA endonuclease activity for a previously described RNA endonuclease (Nob1) and identify a novel AP lyase enzyme (TK0353) that lacks sequence homology to known nucleic acid modifying enzymes.

## INTRODUCTION

With the emergence of easily accessible and inexpensive next-generation sequencing techniques, there has been a surge of publicly available genomes and metagenomic sequencing data. This is especially true for the archaeal domain in which next-generation sequencing has uncovered 10 new major archaeal groups and putative new phyla (1, 2). Importantly, it has been speculated that 30 to 80% of archaeal genes remain unannotated with no assigned gene function and are referred to as ‘dark matter’ (3, 4). Even with the help of manual curation and/or computational approaches to annotate hypothetical proteins, 15 to 40% of these genes still have unknown functions and those with predicted functions are only broadly assigned. Other factors also limit efforts to annotate genes or assign gene function in Archaea, including difficulties in growing and/or culturing archaeal organisms and the lack of high-throughput functional screening methods (5). Therefore, alternative approaches are needed to accelerate the discovery of new archaeal enzyme families and pathways that are distinct from known homologs (6).

High-throughput function-based screening has historically been a powerful approach for identifying new enzymes and assigning gene function. One method employs screening functional (meta)genomic DNA libraries for the expression of new enzyme activities (7–10). Typically, libraries are constructed by cloning fragments of environmental or genomic DNA from a single organism into a backbone vector. The size of the genomic DNA insert depends on the chosen vector. Plasmid vectors accommodate smaller genomic DNA inserts (<10 kb), cosmids and fosmids accommodate larger inserts (typically ∼40 kb), while bacterial artificial chromosomes (BACs) can accommodate the largest inserts (up to ∼200 kb). Cosmid and fosmid vectors are vectors of choice due to the large genomic DNA insert size they accommodate, the ease to manipulate, and the availability of kits to construct and isolate them. Further, fosmids offer an arabinose inducible copy control system, which allows one to tune the fosmid copy number per cell, where a low copy number (1 to 2 copies per cell) ensures the stability of the clone during library construction. Shifting to a higher copy number (50 to 100 copies per cell) enables more robust protein expression during screening.

Once the library is constructed, it is introduced into a screening host (most often E. coli) and transformants are arrayed into plates (10). Each well contains a clone that may express proteins encoded by the foreign genes. Lysates of E. coli clones are then screened for target activities using functional read-out assays utilizing labeled substrates, such as chromogenic substrates or radiolabeled DNA (9–12). Importantly, any organism or metagenomic sample can be used to construct a genomic library using a small amount of collected DNA. Further, the use of E. coli as the expression host allows for screening of genes with desired activities derived from difficult to grow or unculturable organisms.

Functional genomic cosmid libraries have previously been used to identify a variety of novel DNA modifying enzymes from Pyrococcus furiosus, including a novel DNA polymerase, DNA endonuclease, DNA exonuclease, and resolvase (11–14). Metagenomic fosmid libraries have been used to identify novel enzyme activities, including the recent discovery of a novel exosialidase family and a novel DNA polymerase (10, 15). Further, the functional genomics methodology has been previously used to identify esterases, proteases, carbohydrate hydrolases, and lipases from thermophiles (7–9), although the utilization of heterologous DNA promoters by the E. coli host machinery to carry out gene expression is not well understood.

In this work, we adapted the functional genomics workflow to screen for DNA modifying enzymes in the hyperthermophilic archaeon Thermococcus kodakarensis by utilizing a fosmid library in conjunction with capillary electrophoresis (CE) as a high-throughput functional read-out. Capillary electrophoresis has been used to functionally characterize a wide variety of DNA modifying enzymes, including DNA polymerases, ligases, glycosylases, and endonucleases, in a high-throughput manner (16, 17). CE relies upon the use of fluorescently labeled DNA substrates that are separated by charge and size and are visualized by laser excitation using a standard Sanger sequencing instrument. Importantly, assays can be multiplexed using DNA substrates with different fluorophores, allowing for several activities to be screened at once, and 96 reactions can be processed in under 1 h. As a proof-of-principle, we first used the function-based screen to identify the known T. kodakarensis DNA repair enzymes uracil DNA glycosylase (UDG), RNaseH2, and DNA ligase. The screening workflow was then used to uncover a new DNA endonuclease activity for a previously described RNA endonuclease (Nob1) (18). We utilized the function-based screening method to discover a novel AP lyase DNA repair enzyme family in T. kodakarensis (termed 'TK0353') that lacks sequence homology to known DNA repair enzymes and, thus, was not identified using standard bioinformatics methods. TK0353 may play a redundant role in AP site repair in T. kodakarensis. Illumina next-generation sequencing (NGS) was performed on the fosmid library to assess fosmid coverage and aid enzyme identification. Finally, RNA-sequencing was performed to demonstrate expression of T. kodakarensis genes by E. coli host machinery and showed a high fraction of T. kodakarensis fosmid genes were transcribed in E. coli despite differences in promoter sequences and translational machinery. This method presents a simple, high-throughput method for the identification and characterization of novel nucleic acid enzymes in hyperthermophilic archaea and provides a path for uncovering the ‘dark matter’ of Archaea.

## RESULTS

### A high-throughput screening method to discover novel enzymes in Thermococcus kodakarensis.

**FIG 1:**
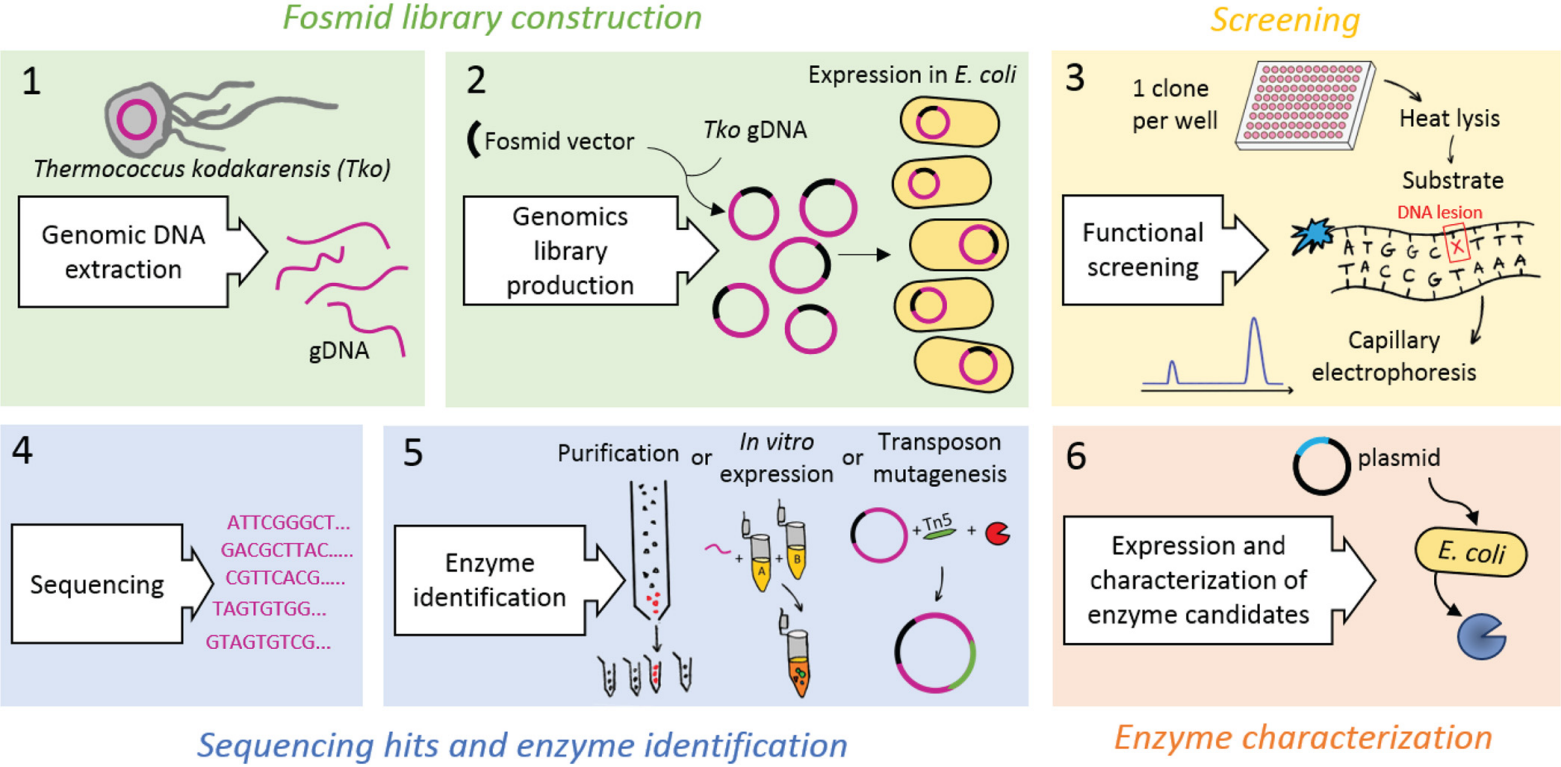
A schematic illustration of the functional genomics workflow to discover new DNA repair enzymes. (i) Genomic DNA is extracted from T. kodakarensis, fragmented into 40 kb pieces, and (ii) cloned into a fosmid vector and propagated in E. coli. (iii) E. coli containing fosmids are arrayed in 384-well plates with one clone per well. E. coli are grown and then a heat lysate is prepared by incubating cells at 80°C for 30 min. E. coli proteins will be inactivated while T. kodakarensis proteins will survive the heat treatment. DNA repair activities are then assayed incubating fosmid lysates with DNA repair substrates and resolved by capillary electrophoresis. (iv) Fosmids that express DNA repair activities are sequenced and annotated. (v) Enzyme activity can be identified by in vitro transcription/translation of candidate proteins, by purification using column chromatography and mass spectrometry, or by transposon insertion mutagenesis. (vi) Once a candidate protein is identified, the protein is expressed recombinantly, purified and its activity further characterized.

We adapted a functional metagenomics approach to screen for DNA modifying activities in T. kodakarensis through the creation of a fosmid library and by utilizing CE as a functional read-out assay. A fosmid library was constructed by cloning 40 kb fragments of T. kodakarensis genomic DNA into a fosmid backbone (Fig. 1, step 1). The fosmids were then used to transform E. coli cells and 1,536 individual clones were arrayed into 384-well plates, theoretically providing ∼30-fold coverage of the entire T. kodakarensis genome (Fig. 1, step 2).

**FIG 2:**
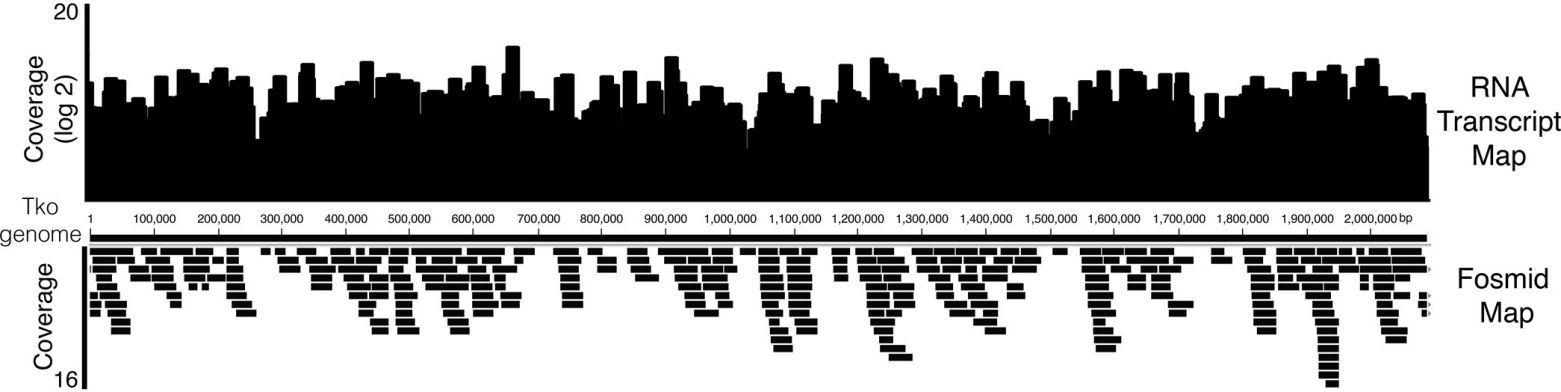
Functional genomics fosmid DNA library and RNA transcripts mapped to the T. kodakarensis genome. A typical functional genomics 384-well plate (Plate NEB260) was sequenced using the plexWell^TM^ 384 Library Preparation kit and Illumina sequencing. Fosmid inserts (bottom black bars; average insert size of 35,233 ± 7,641 bp) were mapped to the T. kodakarensis genome using Geneious software. RNA-seq was conducted on a pooled 384-well plate (Plate NEB260) using NEBNext Ultra^TM^ II RNA Library Prep kit and Illumina Sequencing. RNA transcripts were mapped to the T. kodakarensis genome using HISAT2 and htseq-count using the Galaxy server. Transcript coverage is plotted (log_2_).

To identify active T. kodakarensis DNA modifying enzymes, arrayed E. coli clones were grown in liquid culture to allow for the expression of genes encoded by fosmid. Cells were then heat lysed to inactivate the endogenous E. coli DNA repair enzymes and other proteins while hyperthermophilic T. kodakarensis enzymes remain active (Fig. 1, step 3). Extracts used for screening were prepared by centrifugation of heat lysed cells to separate soluble heat-stable T. kodakarensis proteins from denatured E. coli proteins and cell debris. Extracts were then tested for DNA repair activities using various fluorescently labeled DNA substrates containing site-specific DNA lesions. Extracts from clones encoding DNA repair proteins act on DNA substrates and generate products that can be resolved and analyzed by capillary electrophoresis (CE) (17). Positive “hits” were considered reactions with at least 2-fold higher activity than the background. The ABI 3730xl Genetic Analyzer CE instrument analyzes a 96-well plate in under an hour, providing rapid and high-throughput output. At the same time, fosmids were sequenced and mapped to the T. kodakarensis genome (Fig. 1, step 4, and Fig. 2). Presequencing fosmids in each well permitted rapid identification of candidate genes. This capability differentiates this pipeline from previously described workflows and accelerates the discovery pipeline once an activity is identified.

Once hits were obtained during screening, the fosmid gene that was responsible for the activity was identified. A variety of approaches can be used to identify the gene responsible for the DNA repair activity from the fosmid ORFs (Fig. 1, step 5). In some cases, bioinformatic analysis of the fosmid DNA enabled the identification of gene candidate(s) (as shown below for the Nob1 endonuclease). In other cases, the identification of gene candidate(s) must rely upon activity assays. Different strategies can be used. The DNA modifying enzyme encoded by the fosmid gene can be purified from E. coli by column chromatography and then identified by mass spectrometry using a database of T. kodakarensis proteins (as shown below for TK0353). A second approach expressed each candidate gene using an *in vitro* transcription/translation system (such as NEB PureExpress) and testing for activity (19). A third approach uses Tn5 transposase mutagenesis to interrupt genes by the random insertion of a transposon. Insertion sites that disrupt enzyme activity can be sequenced and mapped to identify the encoding gene (10). Once the gene responsible for the activity has been identified, the enzymes are cloned, recombinantly expressed, and further characterized (Fig. 1 step 6).

### Characterization of a T. kodakarensis fosmid library.

We sequenced a 384-well fosmid library plate (Plate NEB260) in its entirety using plexWell^TM^ 384 Library Preparation kit. The plexWell technology creates individually barcoded libraries for each well that can then be pooled and sequenced by Illumina Sequencing. Sequencing reads were assembled by Geneious Software and mapped to the T. kodakarensis genome (Fig. 2). The average fosmid insert size was 35,233 ± 7,641 bp and pairwise identity was 99.8%. The mean sequencing coverage across the genome was 4.7 ± 3.2 with a maximum coverage of 16. In addition, 13 gaps in coverage ranged in size from 3,512 to 28,084 bp with an average gap length of 13,086 ± 8,786 bp. These data showed that a 384-well fosmid library plate was representative of the majority of T. kodakarensis genes (93%). Gaps in coverage may be due to stochastic fragmentation of cloned fragments during fosmid generation or may also represent toxic sequences that are unable to be cloned in E. coli (20). If required, additional plates can be analyzed for more complete genome coverage. To ensure E. coli expressed T. kodakarensis genes to some level, we performed an RNA-seq experiment to confirm the presence of T. kodakarensis transcripts. Importantly, RNA-seq gene expression analysis of the pooled Plate NEB260 library transcripts showed that the majority of T. kodakarensis genes were transcribed (84% of genes had at least 10 transcript reads).

### Functional genomic screening identifies known DNA repair enzymes.

**FIG 3:**
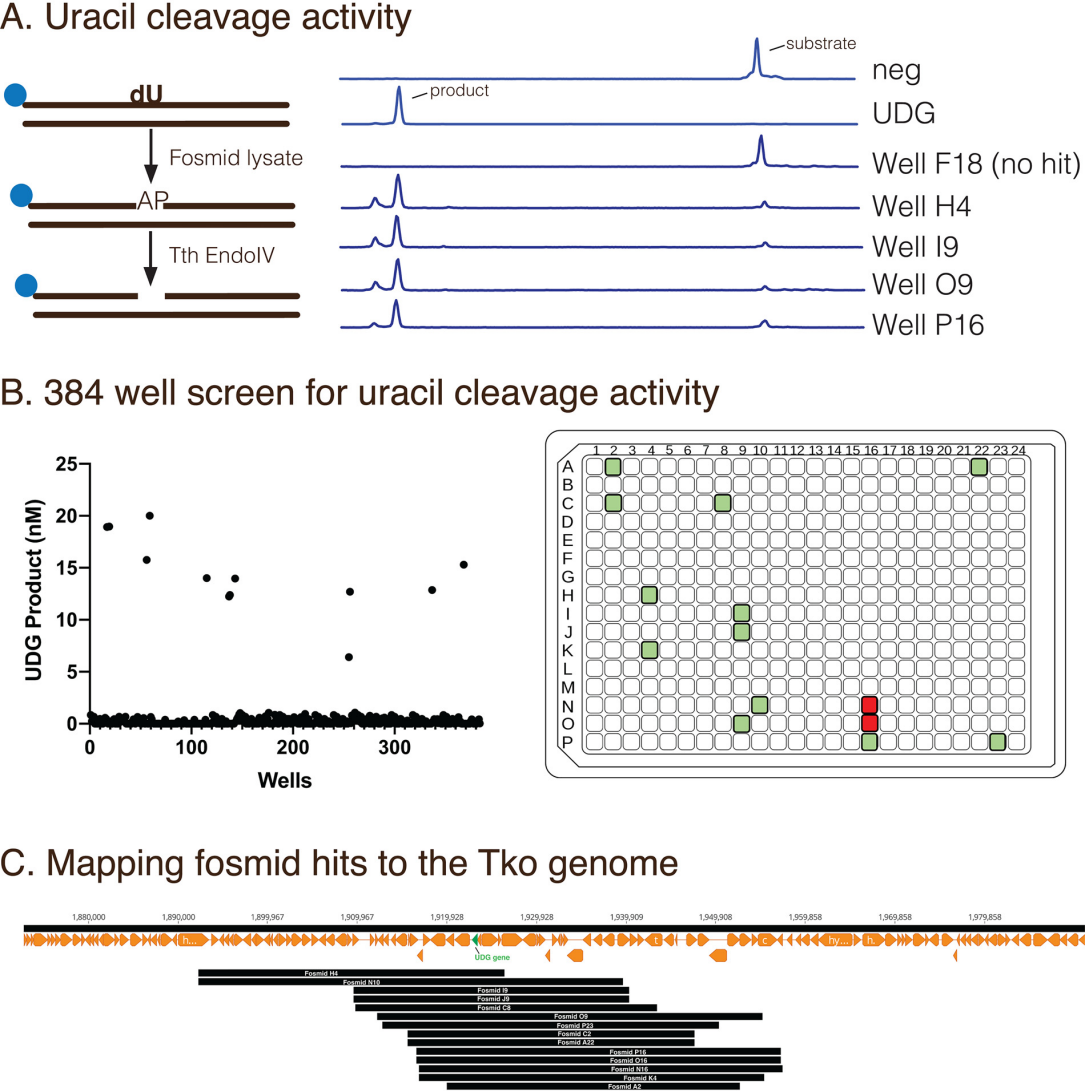
Evaluating the efficiency of functional genomic screening by identification of a known uracil cleavage enzyme. (A) A 5′-FAM labeled DNA substrate containing a dU was incubated with a control fosmid lysate (neg), UDG, or fosmid lysate followed by *Tth* EndoIV to cleave AP sites. Examples of fosmid lysates that contained uracil cleavage activities include Well H4, I9, O9, and P16. (B) A 384-well fosmid plate was screened for UDG activity yielding 12 positive fosmid lysate hits. Colored squares represent 14 fosmid wells that contain the UDG gene and green squares confirm that UDG activity was observed, while red squares lacked detectable UDG activity. (C) Fosmids containing UDG activity mapped to a region in the T. kodakarensis genome containing the UDG gene.

To evaluate the fosmid library for T. kodakarensis enzyme expression in E. coli, we first screened for a well-characterized T. kodakarensis DNA repair enzyme uracil DNA glycosylase (UDG). During DNA repair, UDG cleaves uracil in DNA to leave an abasic site (21, 22). A 5′-FAM-labeled DNA substrate containing a dU was incubated with a control E. coli lysate transformed with pCC1 (neg), purified UDG or fosmid lysate followed by *Tth* EndoIV to cleave AP sites (Fig. 3A). Control lysates lacked detectable UDG activity (Fig. 3B). Lysates from a 384-well fosmid plate were screened yielding 12 positive fosmid lysate hits for UDG activity (Fig. 3B). Examples of fosmid lysates that contained uracil cleavage activities include wells H4, I9, O9, and P16 (Fig. 3A). All 12 fosmid UDG hits mapped to the genomic region containing the UDG gene (TK2143: position 1,922,741 – 1,923,331) (Fig. 3C). Two additional fosmids (O16 and N16) mapped to the UDG region yet lysates did not contain detectable dU removal activity (data not shown).

**FIG 4:**
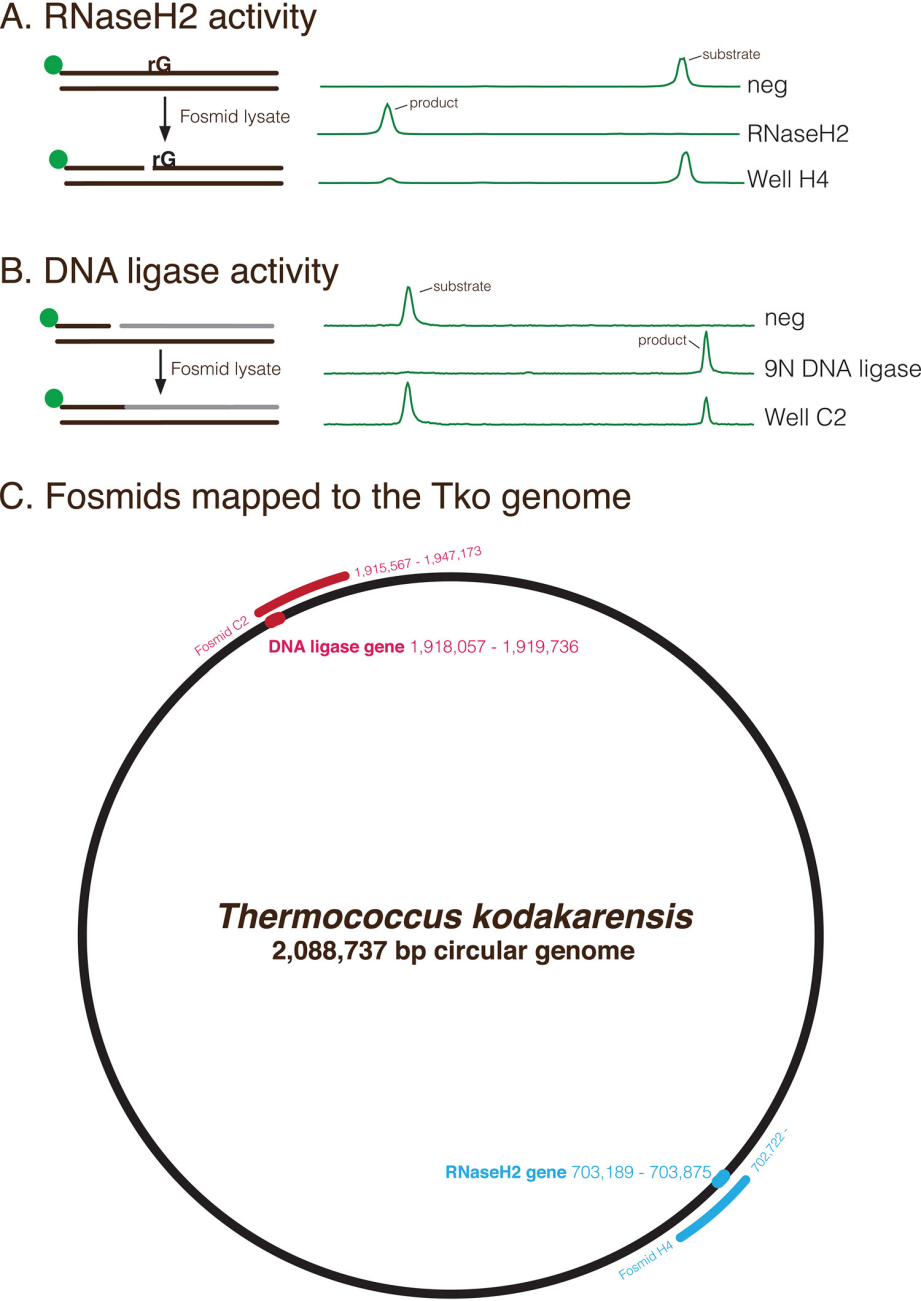
Examples of functional genomics screening to identify RNaseH2, DNA ligase, and Uracil cleavage DNA repair activities. (A) A 5′-MAX labeled DNA substrate containing a single rG was incubated with a control fosmid lysate (neg), purified RNaseH2, or fosmid lysate. Reaction products were resolved by capillary electrophoresis. Lysate from Well H4 contained RNaseH2 activity. (B) A 5′-MAX labeled DNA substrate containing a nick was incubated with a control fosmid lysate (neg), purified 9N DNA ligase, or fosmid lysate. Lysate from Well C2 contained DNA ligase activity. (C) Fosmid inserts were sequenced and mapped to the T. kodakarensis genome. Fosmid H4 sequence starts at position 702,722 bp and overlaps with the RNaseH2 gene (703,189 to 703,875). Fosmid C2 (1,915,567 to 1,947,173) contains the DNA ligase gene (1,918,057 to 1,919,736).

We then screened for other DNA repair activities, including RNaseH2 and DNA ligation. RNaseH2 cleaves 5′ to a ribonucleotide embedded in DNA to initiate repair. RNaseH2 in T. kodakarensis plays a major role in the ribonucleotide excision repair pathway (23). Fosmid lysates were screened for RNaseH2 activity by assessing the ability to cleave a DNA substrate containing a rG. Fosmid H4 lysate contains RNase H2 activity and its sequence (starting at position 702,722) overlaps with TK0805, the gene encoding RNaseH2 (703,189 to 703,875) (Fig. 4A and C). We then screened for the T. kodakarensis DNA ligase (TK2140), an important enzyme in DNA replication and repair (24) that seals nicks in genomic DNA. A nicked DNA substrate was incubated with fosmid lysates and screened for DNA ligase sealing activity. Fosmid C2 lysate contained DNA ligase activity and its sequence (1,915,567 to 1,947,173) overlaps with the DNA ligase gene TK2140 (1,918,057 to 1,919,736) (Fig. 4B and C).

### Functional genomic screening identifies a previously unknown DNA endonuclease activity of T. kodakarensis RNA endonuclease Nob1 (TK0337).

**FIG 5:**
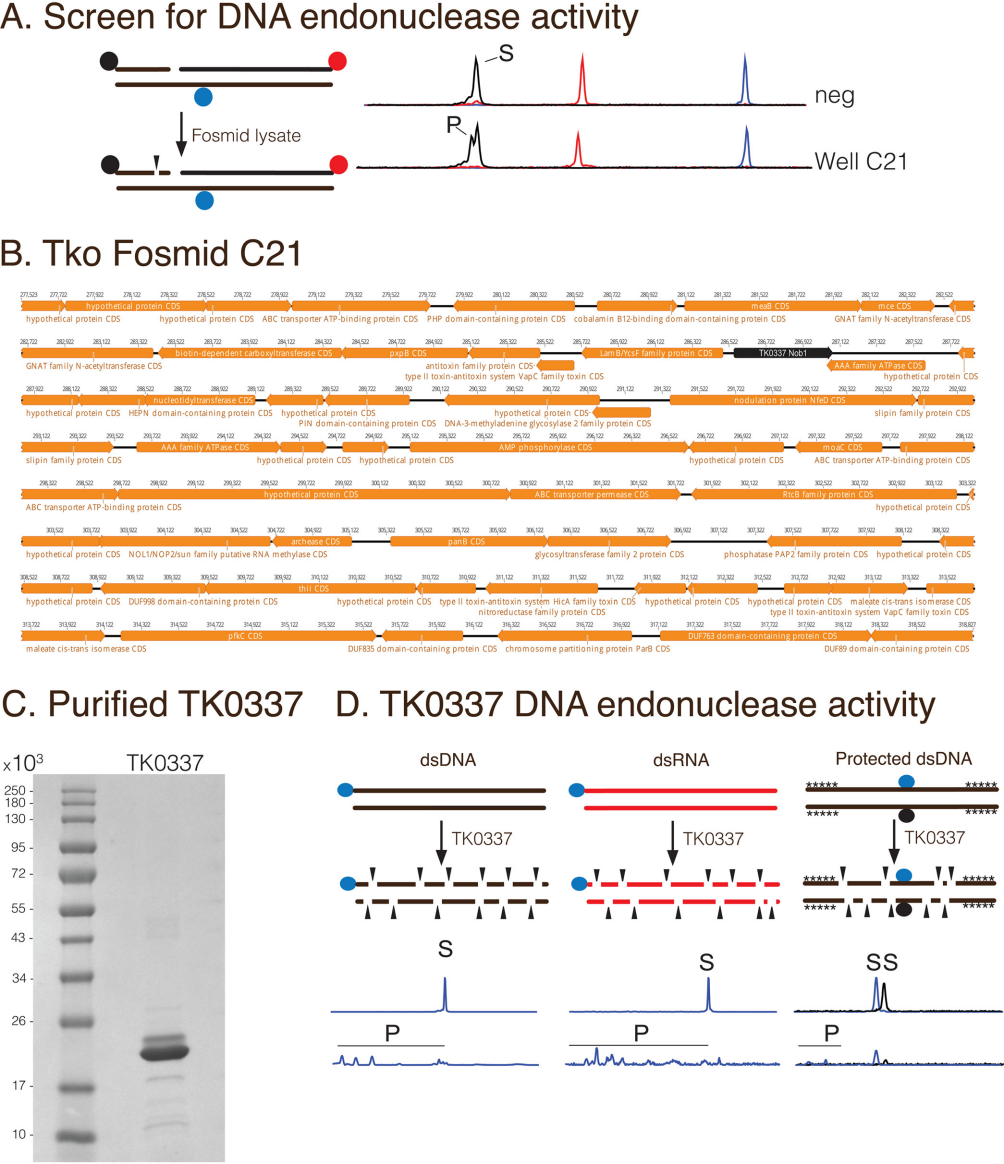
Functional genomic screening identifies a new activity for T. kodakarensis Nob1 RNA endonuclease (TK0337). (A) The T. kodakarensis library was screened for DNA endonuclease activity by incubating a fosmid lysate with a DNA substrate labeled with 5′-TAM (black circles), 3′-ROX (red circles), and internal FAM (blue circles). Reaction products were resolved by capillary electrophoresis. The substrate was incubated with a control fosmid lysate (neg) or fosmid lysates. Lysate from Well C21 contained endonuclease activity on the 5′-TAM labeled strand (black) as indicated by an arrow. (B) The fosmid C21 was mapped to the T. kodakarensis genome using Geneious software and the insert region is shown. (C). TK0337 (20.8 kDa) was expressed recombinantly in E. coli and purified as described in Materials and Methods. (D) Labeled substrates were incubated with purified recombinant TK0337 Nob1 and CE analysis reveals endonuclease activity on dsDNA, dsRNA, and dsDNA protected with phosphorothioate bonds (shown as *). As controls, substrates were incubated with no enzyme. Cleavage sites are shown schematically by arrows.

Functional genomic screening for known DNA repair enzymes confirmed that the pipeline is an efficient and effective method for detecting enzymes that act upon DNA. As such, we next sought to identify new DNA modifying enzymes using this method. To screen for novel DNA endonucleases, we incubated fosmid lysates with a fluorescently labeled DNA substrate with an internal nick (Fig. 5A). The substrate was incubated with fosmid lysates and screening identified one well (C21) containing endonuclease activity at nicked sites (Fig. 5A). Control fosmid lysates lacked detectable activity (Fig. 5A). Fosmid C21 was sequenced and mapped to the T. kodakarensis genome at positions 277,523 to 318,827 (Fig. 5B). One of the genes encoding fosmid C21 was TK0337, annotated as the RNA Nob1 endonuclease. Nob1 was previously shown to be an RNA endonuclease and functions in the 18S rRNA maturation pathway (18). To further characterize TK0337, we recombinantly expressed and purified TK0337 in E. coli (Fig. 5C). While the denaturing gel showed two bands for purified TK0337 (Fig. 5C), mass spectrometry analysis revealed both bands were TK0337, and a degraded form resulted from the heating of the sample before PAGE (data not shown) (25). We incubated purified TK0337 with a variety of DNA and RNA substrates and observed cleavage of dsDNA and dsRNA (Fig. 5D) as well as ssDNA and ssRNA (data not shown) by TK0337. Further, we incubated TK0337 with a dsDNA duplex in which all 5′ and 3′ ends were protected by phosphorothioate bonds and observed cleavage (Fig. 5D), suggesting TK0337 acts as a DNA endonuclease. These data demonstrate a previously undescribed biochemical aspect of Nob1 and suggested it also acts as a DNA endonuclease in addition to its previously characterized role in rRNA processing.

### Functional genomic screening identifies a novel DNA repair AP lyase family from Thermococcus kodakarensis.

**FIG 6:**
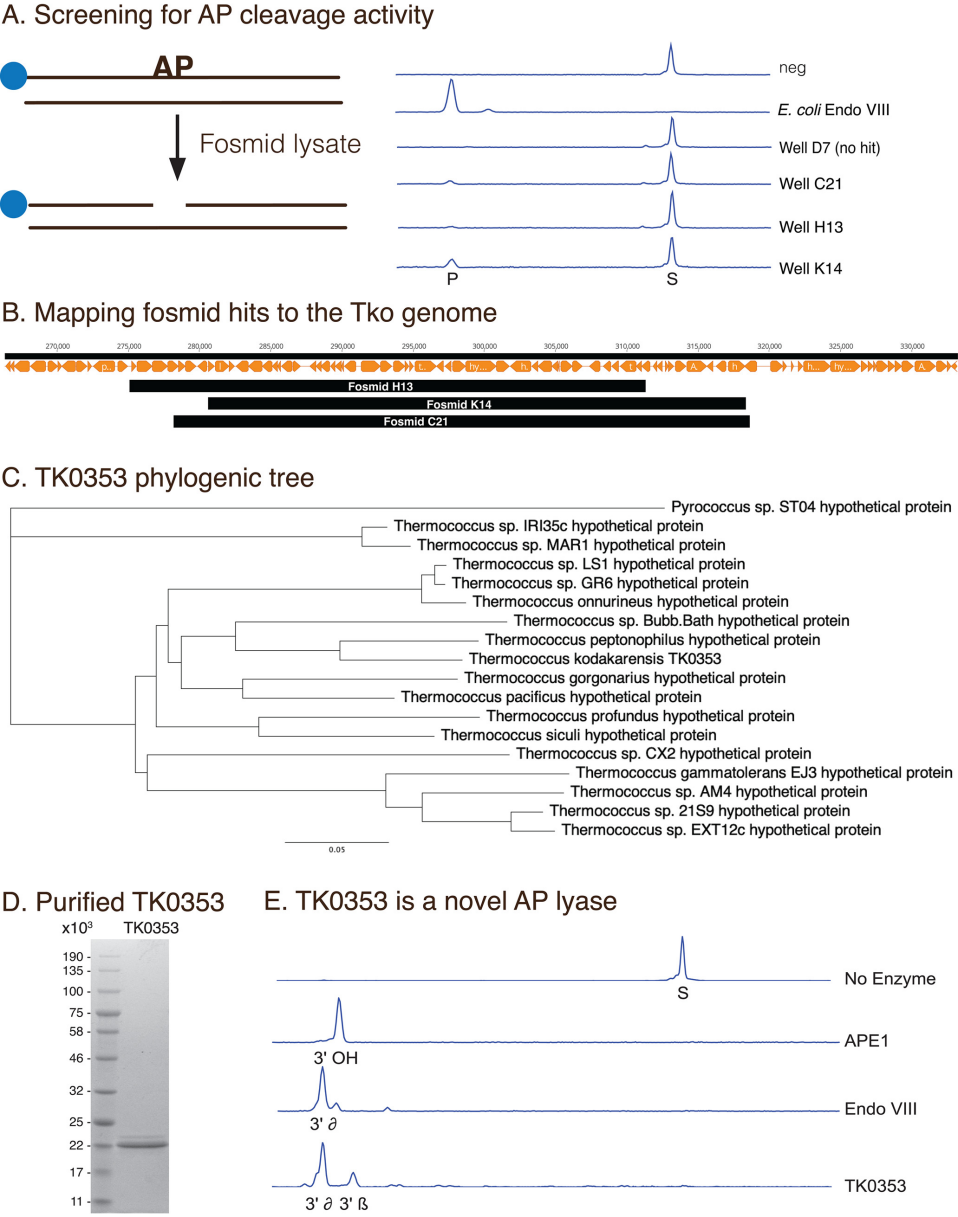
Functional genomics screening identifies a novel *Thermococcus* AP lyase family. (A) A 5′-FAM-labeled DNA substrate containing an AP site substrate was incubated with a control fosmid lysate (neg), Endonuclease VIII, or fosmid lysate. Reaction products were resolved by capillary electrophoresis. Lysate from well D7 lacked AP cleaving activity while wells C21, H13, and K14 contained AP cleavage activity. (B) The fosmid inserts from Fosmid H13, K14, and C21 were sequenced and mapped to the T. kodakarensis genome using Geneious software. (C) TK0353 homologs are shown as a phylogenic tree. (D) TK0353 was expressed recombinantly in E. coli and purified as described in Materials and Methods. (E) FAM-labeled DNA substrate containing an AP site was used to characterize AP cleavage activities using purified enzymes. AP endonuclease (APE) cleaves AP sites to produce a 3′-OH and 5′-sugar fragment. E. coli endonuclease VIII and TK0353 are AP lyases that cleave AP sites via beta and delta elimination.

AP sites are one of the most abundant DNA lesions and are formed by spontaneous hydrolysis of the N-glycosidic bond or by DNA repair glycosylases. Unrepaired AP sites can be both mutagenic and cytotoxic to a cell. As such, organisms encode AP cleavage enzymes to initiate DNA repair. To investigate the breadth of AP cleavage enzymes encoded by T. kodakarensis, the fosmid library was screened for AP cleavage activity by incubating lysates with a fluorescent DNA substrate containing an AP site (Fig. 6A). In total, three wells (H13, K14, C21) contained AP site cleavage activity (Fig. 6A). The fosmid sequences from H13, K14, and C21 all mapped to an overlapping 30 kb region (from 218,358 to 311,780 bp) of the T. kodakarensis genome from 218,358 to 311,780 bp (Fig. 6B). This region contained 51 open reading frames (ORFs), including a variety of metabolic and nucleic acid enzymes and hypothetical proteins. Notably, this region lacks a known or predicted AP cleavage enzyme. To identify and isolate the enzyme responsible for the AP activity of these clones, we grew 1 L of E. coli transformed with the fosmid K14 and purified proteins containing AP cleavage activity by column chromatography. A proteomics approach was then used to identify the proteins present in active fractions. Most proteins identified by mass spectrometry were from E. coli; however, three peptides mapped to the hypothetical protein TK0353 sequence (Table S1 and Fig. S1). The phylogenic analysis found that TK0353 homologs were limited to *Thermococcus* species and one *Pyrococcus* strain as shown in a phylogenic tree (Fig. 6C). TK0353 homologs share high sequence similarity (Fig. S2) yet diverge from any other sequences in public databases and show extremely low sequence homology to known AP lyases.

To biochemically characterize TK0353, the gene was cloned, expressed and recombinant TK0353 was purified (Fig. 6D). Recombinant TK0353 cleaved DNA containing an AP site (Fig. 6E) confirming that it was responsible for the activity observed during screening. AP cleavage enzymes fall into two main classes: AP endonucleases and AP lyases. AP endonucleases are metal ion-dependent, typically requiring a divalent Mg^2+^ ion and cleave AP sites to leave a gap in the DNA backbone with a 3′-OH and 5′-deoxyribose phosphate (dRP) group (Fig. 6E; APE1). Conversely, AP lyases are metal ion independent and cleave the DNA backbone via β-elimination and further δ-elimination reaction, leading to a gap in the DNA backbone with a 5′ phosphate and either a 3′ phosphate or 3′ α, β-unsaturated aldehyde (Fig. 6E; endonuclease VIII). Our initial cleavage experiment suggested TK0353 is an AP lyase generating 3′ β and δ-elimination products. To further characterize TK0353 and confirm an AP lyase mechanism, two experiments were performed. First, we assessed the ability of TK0353 to cleave DNA containing the AP site analog tetrahydrofuran (THF). THF lacks the 1’OH present on the canonical AP site, and therefore cannot undergo a β− and further δ-elimination. Thus, a THF site cannot be cleaved by an AP lyase but can be cleaved by an AP endonuclease. TK0353 does not cleave a THF site, while a known AP endonuclease (APE1) can readily cleave a THF site (Fig. S3). Next, we assessed the metal ion dependence of TK0353 on AP site cleavage by performing cleavage reactions in the presence or absence of Mg^2+^ in addition to preincubating TK0353 with EDTA to remove any prebound metal. TK0353 displayed AP cleavage activity under all conditions tested, suggesting it is a metal ion-independent AP lyase (Fig. S4).

## DISCUSSION

A major limitation to discovering novel enzyme families is the reliance on bioinformatics to find closely related protein homologs. Enzyme discovery using bioinformatics requires some previous knowledge of protein sequence and function thereby limiting the identification of completely new scaffolds that lack amino acid similarity with known proteins. The functional genomics approach described here complements sequence-based bioinformatic homology searching. Functional genomic screening does not require prior knowledge of protein sequence or functional information. In addition, because less than 1% of organisms can be cultivated in the lab, expressing heterologous genes in E. coli fosmid libraries expands access to genetic content from any cultivated single organism or environmental DNA source (26). This platform for enzyme discovery allows a more complete understanding of the diversity of enzymes and pathways that underlie core cellular processes and opens discovery areas.

In this study, screening the T. kodakarensis fosmid library for enzyme activity on a DNA substrate identified a new activity for the RNA endonuclease Nob1 (TK0337). Nob1 from Pyrococcus horikoshii is a Mn^2+^-dependent RNA endonuclease that processes pre-rRNA sequences at the predicted 3′ end of 16S rRNA during ribosomal biogenesis (18). In this study, our fosmid screen revealed a similar activity on the 3′-end of a DNA substrate initiating at a nick, while purified Nob1 from T. kodakarensis showed endonuclease activity on both DNA and RNA substrates. The DNA and RNA endonuclease activities may reflect a redundant role for Nob1 in both rRNA processing and DNA repair or metabolism. Another novel enzyme found via the functional genomics platform was TK0353 that represents a new family of AP lyases found only in *Thermococcus* and one *Pyrococcus* strain. TK0353 would not easily be identified by bioinformatic analysis as a DNA repair enzyme because it was annotated as a hypothetical protein and diverged from known DNA repair proteins. Because DNA damage occurs often in high-temperature environments, TK0353 may play a backup role in processing AP sites to complement the essential AP lyase in T. kodakarensis, endonuclease IV (TkoEndoIV) (27). Future studies will characterize TK0353 biochemistry in molecular detail.

One potential limitation of expressing heterologous genes in fosmid libraries may be poor expression in E. coli due to differences in native promoter sequences, translation initiation elements, or codon usage (8, 9). The poor expression would mean some activities could be overlooked when using this functional genomics methodology. Previous approaches proposed increasing transcription of heterologous genes in fosmid libraries by coexpressing heterologous sigma factors from other bacterial species to direct E. coli RNA polymerase to a diversity of bacterial promoters in the fosmid library (28). Alternatively, to improve heterologous gene expression of thermophilic proteins, Thermus thermophilus was used as a host to screen thermophilic esterases from hot springs metagenomic fosmid libraries (7). In this study, we directly measured the transcription of heterologous genes in a representative E. coli fosmid library plate. RNA-seq data showed a remarkably high fraction of T. kodakarensis fosmid genes that were transcribed in E. coli. The RNA-seq data provides confidence that a majority of T. kodakarensis genes can be expressed heterologously in E. coli. It is unclear if the expression of T. kodakarensis genes in E. coli is due to usage of archaeal promoters by E. coli transcriptional machinery, spurious E. coli transcription at nonpromoter sites (i.e., intragenic initiation), or as a result of transcriptional read-through of vector promoters. Computational analysis of intrinsic archaeal promoter elements and ribosome binding sites predicted that 30 to 50% of euryarchaeal genes may be transcribed in E. coli (29). Thus, simply characterizing the transcriptome of fosmid libraries can give assurance of the extent of functional coverage of the included genes and indicate whether one of these alternative approaches is necessary for the expression of most proteins. It is important to note, that sequence analysis of several hits with UDG activity revealed that the UDG gene is located within the middle of the fosmid insert, ∼20 kb away from the vector backbone, suggesting that transcription read-through of vector promoters is unlikely at least for some fraction of transcripts. Future studies will continue to define archaeal cis and trans transcriptional elements that influence heterologous gene expression in E. coli.

In addition to bioinformatic and gene expression hurdles, the lack of readily available high-throughput functional assays has limited the discovery of novel enzymes. Previously used functional genomic read-out assays to look for DNA modifying enzymes rely upon radiolabeled DNA substrates to detect enzyme activity (11–14). While this method has aided in the detection of an archaeal DNA polymerase, endonuclease, exonuclease, and resolvase, these substrates cannot be multiplexed and analyzed in a high-throughput fashion. Here, we described the use of CE as a high-throughput functional read-out assay to identify novel DNA repair enzymes in conjunction with NGS sequencing. This optimized workflow enables visualization of a variety of different DNA modifying activities concurrently and rapid downstream enzyme identification. While in this work we show the ability to detect DNA cleavage and joining activity from enzymes in the fosmid lysates, CE can resolve both DNA and RNA oligonucleotides, therefore, one, in theory, could detect any enzyme activity that modifies DNA or RNA.

Continued improvement in computational methods, high-throughput assay design, and functional genomics screens will accelerate the potential for new enzyme discoveries. New highly multiplexed assays can be designed to further increase the number of clones screened to compensate for the relatively low hit rate in some libraries. Multiple fluorescently labeled substrates can be multiplexed in a single reaction allowing concurrent analysis of a wide variety of DNA enzyme activities, including DNA polymerization, exonuclease digestion, DNA ligation, and DNA cleavage (17). Even higher throughput assays include emulsion-based assays that encapsulate single metagenomic clones into droplets (30). Millions of encapsulated clones can then be screened using fluorescent substrates and fluorescence-activated cell sorting to identify desired activities.

With these advances, functional genomic approaches will be used to address new biological questions in T. kodakarensis and other hyperthermophiles and organisms that are difficult to cultivate. Specifically, there is the opportunity to screen for important enzyme activities that are “missing” from some genome annotations. but may simply be present in genes too divergent from characterized proteins to reliably annotate based on primary sequence homology alone. For example, T. kodakarensis genome annotation lacks key enzymes found in bacteria and eukarya, such as MutT that hydrolyzes oxidized nucleotides (8-oxo-dGTP), MutY which repairs dA:8-oxo-dG mismatches, or a Y-family DNA polymerase that performs translesion DNA synthesis. As shown in this study, functional genomics approaches will continue to uncover new enzyme families and broaden understanding of the diversity of enzymes and pathways for core biological functions.

## MATERIALS AND METHODS

### Preparation of DNA substrates.

All enzymes used in this study were from New England Biolabs (Ipswich, MA) unless otherwise stated. The oligonucleotides used for capillary electrophoresis were from Integrated DNA Technologies (Coralville, IA). Oligonucleotide sequences are provided in Table 1.

**TABLE 1:** DNA Oligonucleotides used in this study

Substrate	Sequence
5′MAX rG 50-mer	5′ MAX-GGG AGC GTC GAC AGC TTG GAT **rG**AG TGC CAC TTG TCT ACG GCT ATG CCT TA-3′
50-mer complement	5′-TAA GGC ATA GCC GTA GAC AAG TGG CAC TCA TCC AAG CTG TCG ACG CTC CC-3′
5′FAM 3′ROX dU 60-mer	5′-FAM-TGG AGA TTT TGA TCA CGG TAA CC**dU** ATC AGA ATG ACA ACA AGC CCG AAT TCA CCC AGG AGG-3′-ROX
5′FAM 3′ROX THF 60-mer	5′-FAM-TGG AGA TTT TGA TCA CGG TAA CC**dSpacer** ATC AGA ATG ACA ACA AGC CCG AAT TCA CCC AGG AGG-3′-ROX
60-mer Complement	5′-CCT CCT GGG TGA ATT CGG GCT TGT TGT CAT TCT GAT GGG TTA CCG TGA TCA AAA TCT CCA-3′
DNA endonuclease Upstream	5′-NED-CG CAC CCT TAC CAC CAA GAC AGG ATC GTC CTT GC-3′
DNA endonuclease Downstream	5′-TGA TCA TGC ATC GTT CCA CTG TGT CCG CGA CAT CTA CGT C-ROX-3′
DNA endonuclease Splint	5′ -GAC GTA GAT G/iFluorT/C GCG GAC ACA GTG GAA CGA TGC ATG ATC AGC AAG GAC GAT CCT GTC TTGG TGG TAA GGG TGC GC-3′
5′-VIC-DNA ligation acceptor	5′-VIC-CGC CAG GGT TTT CCC AGT CAC GAC-3′
Ligation donor	5′-P-GTT GTA AAA CGA CGG CCA GTG CCA AGC TTG-3′
DNA ligation template	5′-CAA GCT TGG CAC TGG CCG TCG TTT TAC AAC GTC GTG ACT GGG AAA ACC CTG GCG-3′
5’FAM 50-mer DNA Endonuclease	5′-FAM-AGT GAA TTC GAG CTC GGT ACC CGG GGA TCC TCT AGA GTC GAC CTG CAG GC
50-mer DNA Endonuclease Com	GCC TGC AGG TCG ACT CTA GAG GAT CCC CGG GTA CCG AGC TCG AAT TCA CT
5’FAM 50-mer RNA Endonuclease	5′-FAM-rArGrU rGrArA rUrUrC rGrArG rCrUrC rGrGrU rArCrC rCrGrG rGrGrA rUrCrC rUrCrU rArGrA rGrUrC rGrArC rCrUrG rCrArG rGrC
50-mer RNA Endonuclease Com	rGrCrC rUrGrC rArGrG rUrCrG rArCrU rCrUrA rGrArG rGrArU rCrCrC rCrGrG rGrUrA rCrCrG rArGrC rUrCrG rArArU rUrCrA rCrU
39mer protected FluoroT DNA	C*A*T* G*A*G GTA A/iFluorT/C TAC GTG CTG TTA TGT TGT CCA T*C*A* G*G*C
39mer protected TAM DNA	G*C*C* T*G*A TGG ACA ACA TAA CAG CAC GTA GA/i6-TAMN/ TAC C*T*C* A*T*G

The oligonucleotide substrate used to screen ribonucleotide cleavage activity was prepared by annealing 1 μM the fluorescently labeled 5′-MAX rG 50-mer oligonucleotide to 1.25 μM the 50-mer complementary oligonucleotide in 1× annealing buffer (10 mM Tris-HCl pH 7.5 and 100 mM NaCl) at 85°C for 5 min and allowing the reactions to slowly cool to room temperature. The oligonucleotide used to screen for uracil cleavage activity was prepared by annealing 1 μM the 5′-FAM 3′-ROX dU 60-mer labeled lesion containing oligonucleotide to 1.25 μM the 60-mer complementary oligonucleotide in 1× annealing buffer at 85°C for 5 min and allowing the reactions to slowly cool to room temperature. For uracil removal activity assays, the complementary oligonucleotide contained a G across from the dU lesion. AP site DNA was freshly prepared before each experiment by incubation of 100 nM dsDNA containing dU:G with 1 unit of uracil DNA glycosylase (UDG; New England Biolabs) in 1× Thermopol buffer (20 mM Tris-HCl, 10 mM _[NH4]2_SO_4_, 10 mM KCl, 2 mM MgSO_4_, pH 8.8 at 25°C) in a 100 μL reaction for 30 min at 37°C. To confirm conversion of all dU sites to AP sites, AP site DNA was incubated with Thermus thermophilius Endonuclease IV 1 (*Tth* EndoIV, New England Biolabs) at 37°C for 5 min. For DNA ligation assays, 1 μM 5′-VIC-DNA ligation acceptor oligonucleotide was annealed with 1.25 μM ligation donor and 1.5 μM DNA ligation template as described above. For DNA endonuclease assays a 3-color nicked DNA construct was created in which 1 μM DNA endonuclease upstream oligonucleotide was annealed with 1 μM DNA endonuclease downstream oligonucleotide and 1 μM DNA endonuclease Splint as described above. For T. kodakarensis Nob1 characterization assays, 1 μM 50-mer DNA or 50-mer RNA was annealed to 1.25 μM the DNA or RNA complement as described above and 1 μM protected FluorT-39-mer DNA was annealed to 1 μM protected TAM 39-mer protected DNA as described above.

### Construction of a T. kodakarensis fosmid library.

Genomic DNA (gDNA) was purified from T. kodakarensis [ATCC (Manassas, VA)] and was used to generate a fosmid library containing 35 to 40 kb inserts. The T. kodakarensis gDNA fosmid libraries were constructed by Bio S&T (Montreal, Canada) using the pCC1FOS cloning vector. Bio S&T performed a large-scale transfection of EPI300-T1^R^ cells and arrayed 1,536 clones into glycerol in four 384-well plates to permit their storage at –80°C.

### Sanger and next-generation sequencing of the fosmid library.

To determine the genes encoding each fosmid clone both Sanger and Illumina sequencing were performed. For Sanger sequencing, individual isolated fosmids were sequenced using forward and reverse primers annealed to the 8 kb vector backbone: pCC1 Forward Sequencing Primer 5′-GGA TGT GCT GCA AGG CGA TTA AGT TGG-3′ and pCC1 Reverse Sequencing Primer 5′-CTC GTA TGT TGT GTG GAA TTG TGA GC-3′. Sanger sequencing captures the sequence for the outermost portion of T. kodakarensis genomic DNA fosmid insert, but not the entire fosmid insert. Sequences were then mapped to the T. kodakarensis reference genome (GenBank accession number: NC_006624) to identify the full genomic region present in each clone.

In addition to Sanger sequencing, a high-throughput library construction method to prepare Illumina sequencing libraries from a multiplexed 384-well fosmid library plate (Plate NEB260) was also utilized. This method utilizes a transposon-based barcode insertion strategy and PCR amplification to create Illumina libraries. For Illumina sequencing, fosmids were isolated by Bio S&T (Montreal, Canada). Isolated fosmid DNA was quantified using the Quant-iT™ PicoGreen™ dsDNA assay kit (ThermoFisher Scientific, Waltham, MA) according to the manufacturer. After DNA quantitation, multiplexed sequencing libraries were constructed using the plexWell^TM^ 384 Library Preparation kit (seqWell, Danvers, MA) for Illumina Sequencing Platforms following the manufacture’s protocol. Briefly, using a transposase, 96 DNA samples were barcoded with different i-7-barcoded adapters. The barcoded samples were pooled into one tube and an i-5-barcoded adapter was added. The libraries were amplified and purified. The quality of each library was assessed on an Agilent Tapestation (Agilent Technologies, Santa Clara, CA) using the High Sensitivity D5000 kit. Samples of 384 DNA libraries were pooled and run on an Illumina MiSeq instrument (Illumina, San Diego, CA). After sequencing, each DNA fosmid was assembled by seqWell. The pCC1FOS vector backbone was trimmed from each sequence and the resulting fosmid insert sequences were mapped back to the T. kodakarensis genome.

### RNA-seq analysis of T. kodakarensis fosmid library.

In parallel to fosmid DNA sequencing, total T. kodakarensis gene expression from fosmids was analyzed by RNA-seq. E. coli fosmid plate NEB260 was grown as described below and pooled. RNA was extracted using Monarch Total RNA Miniprep kit, including DNase I as directed by the manufacturer (New England Biolabs, Ipswich, MA) and quantified by Nanodrop. NEBNext Ultra II Directional RNA Library Prep kit (NEB number E7760), NEBNext rRNA Depletion kit (New England Biolabs number E7850), and NEBNext Multiplex Oligos for Illumina (Set 2; New England Biolabs number E7500) were used for NGS library prep.

E. coli rRNA was depleted using the NEBNext rRNA depletion kit (bacterial) (New England Biolabs number E7850) as described by the manufacturer. Briefly, 1 μg of RNA was diluted in 11 μL of nuclease-free water and mixed with 2 μL of rRNA Depletion Solution and 2 μL of Probe Hybridization buffer followed by the heating 2 min at 95°C followed by cooling ramp down to 22°C at 0.1°C/sec and then a 5 min hold at 22°C. Subsequently, RNase H Digestion was performed by mixing 15 μL of the sample with 1 μl nuclease-free water, 2 μl of RNase H reaction buffer and 2 μl of NEBNext Thermostable RNase H followed by incubation at 50°C for 30 min. After 30 min, 5 μl of DNase I reaction buffer, 2.5 μl of DNase I, and 22.5 μL of nuclease-free water were added and the sample was incubated for 30 min at 37°C.

After sample purification by beads (NEBNext RNA Sample Purification Beads, NEB #E7104S, 1.8× beads), 5 μL sample in nuclease-free water was incubated with 1 μL of random primers and 4 μL NEBNext First Strand Synthesis reaction buffer at 94°C for 15 min. The fragmented and primed RNA was then mixed with 8 μL NEBNext Strand Specificity Reagent and 2 μL of NEBNext First Strand Synthesis Enzyme Mix and incubated for 10 min at 25°C followed by 15 min at 42°C and then 15 min at 70°C followed by a hold at 4°C. This incubation was followed by second-strand cDNA synthesis by the addition of 8 μL of NEBNext Second Strand Synthesis Reaction Buffer with dUTP mix, 48 μL of nuclease-free water, and 4 μL of NEBNext Second Strand Synthesis Enzyme Mix followed by incubation for 1h at 16°C. After cDNA synthesis, a bead purification (1.8×) was conducted, and 50 μL of DNA was eluted in 0.1× TE buffer. cDNA library ends were converted by adding 7 μL of NEBNext Ultra II End Prep Reaction buffer and 3 μL of NEBNext Ultra II End Prep Enzyme Mix followed by incubation for 30 min at 20°C, 30 min at 65°C, and hold at 4°C. Adaptor ligation was then conducted by adding 2.5 μL of the 5-fold diluted adaptor, 1 μL of NEBNext ligation enhancer, and 30 μL of NEBNext Ultra II ligation Master Mix with incubation at 20°C for 15 min. USER Enzyme (3 μL) was then added and the reaction mixture was incubated at 37°C for 15 min. The library was then purified with 0.9× DNA purification beads. Finally, 15 μL of adaptor-ligated DNA, 25 μL of NEBNext Ultra II Q5 Master Mix, 5 μL of Universal PCR Primer/i5 Primer, 5 μL of Index (X) Primer/i7 Primer (NEBNext Multiplex Oligos for Illumina Set 2, NEB number E7500) were mixed for PCR Enrichment. The PCR was conducted under the following conditions: initial denaturation at 98°C for 30 s; denaturation at 98°C for 10 s, annealing/extension at 65°C for 75 s with 16 cycles; final extension at 65°C for 5 min with 12 cycles. After PCR enrichment, 0.9× beads were used to purify DNA and 0.1× TE was used to elute DNA for Assess Library Quality on Agilent 2100 Bioanalyzer (2100 Expert Software, Agilent, Santa Clara, CA). Sample concentrations were confirmed by Qubit DNA HS Assay (Thermo Fisher Scientific).

The libraries were sequenced on a NextSeq instrument (Illumina, San Diego, CA) using the settings Mid Output 2 × 75 with a 6 bp index at a 3 nM concentration with 5% PhiX spiked in to increase diversity. Sequencing reads were uploaded to Galaxy (Galaxy) and adapter sequences were trimmed using Trimmomatic (Galaxy). The HISAT2 tool (Galaxy) was then used to align the reads to the T. kodakarensis FASTA file (NC_006624). The HISAT2 output BAM file was then mapped against the T. kodakarensis GFF (GCF_000009965.1) file using the HT-Seq (Galaxy) to map reads per gene.

### Capillary electrophoresis screening assays utilizing fosmid lysates.

Each well in a 384-well plate of library clones was grown in 4 mL LB medium containing chloramphenicol (12.5 mg/mL) and 1× inducing solution in 24-deep well plates at 37°C overnight. The cells were pelleted at 3800 rpm for 10 min. The supernatant was removed, each pellet was suspended in 200 μL of 20 mM Tris-HCl pH 7.5 containing 50 mM NaCl, transferred to a 96-well plate, and heat-treated at 80°C for 20 min to lyse the cells and inactivate E. coli proteins. Following heat treatment, the lysate was centrifuged again at 3800 rpm for 10 min. The soluble fraction was removed, transferred to a fresh 96-well plate, and a second heat treatment was performed at 80°C for 20 min was performed to ensure the inactivation of E. coli proteins (especially E. coli nucleases). Clarified lysate was then used in the function-based screening workflows.

To screen the fosmid lysates for DNA modifying activities, a 20 mL reaction mixture containing 20 nM labeled substrate, 5 μL clarified heat-treated E. coli cell extract, and 1× ThermoPol buffer (20 mM Tris-HCl, 10 mM (NH_4_)_2_SO_4_, 10 mM KCl, 2 mM MgSO_4_, pH 8.8 at 25°C) was incubated at 65°C for 30 min. Oligonucleotide substrates are listed in Table 1. All reactions were halted with the addition of 10 μL of 50 mM EDTA. Samples were analyzed by capillary electrophoresis using an ABI3730 xL DNA analyzer (17).

To screen for polymerases, endonucleases, and exonucleases, a 20 μL reaction mixture containing 50 nM substrate (Table 1), 10 μL clarified heat-treated E. coli cell extract, 1× NEBuffer 2 (50 mM NaCl, 10 mM Tris-HCl, 10 mM MgCl_2_, 1 mM DTT, pH 7.9 at 25°C), and 200 μM deoxynucleotide solution mix (dNTP solution mix, New England Biolabs) was incubated at 45°C for 1 h. A positive control was performed using 2.5 units/mL of Bacillus stearothermophilus DNA polymerase, full-length (Bst DNA polymerase, full-length, New England Biolabs). The reactions were diluted at 1:100 and analyzed by capillary electrophoresis.

### Identification of TK0353 using column fractionation.

Column chromatography was used to purify and identify the AP lyase protein encoded by fosmid K14. E. coli transformed with fosmid K14 was grown in 0.5 L LB medium supplemented with chloramphenicol (12.5 mg/mL final concentration) and 1× l-arabinose inducing solution (Lucigen, Middleton, WI) overnight at 37°C. Cells were harvested by centrifugation and the pellet was suspended in 20 mM Tris-HCl pH 7.5, 300 mM NaCl and lysed using a constant cell disruptor (Constant Systems LTD, Northants, UK). The cell lysate was heated to 80°C for 20 min in a water bath and clarified by centrifugation. The clarified lysate was loaded onto a HiPrep™ 16/10 DEAE column (GE Lifesciences, Pittsburgh, PA) to remove DNA contamination. The flowthrough was collected, diluted to 20 mM Tris-HCl, pH 7.5, 50 mM NaCl, and loaded onto a HiPrep 16/10 Heparin column (GE Lifesciences, Pittsburgh, PA). Fractions were eluted using a 50 – 1000 mM NaCl gradient. Fractions containing AP lyase activity were identified using the AP lyase activity assays as described above. These fractions were diluted to 20 mM Tris-HCl, pH 7.5, 50 mM NaCl and loaded onto a HiPrep 16/10 Source Q column. Bound proteins were eluted using a 50 to 1000 mM NaCl gradient. Fractions containing AP lyase activity were pooled and concentrated to 0.2 mg/mL. To identify proteins in the pooled samples, total protein was digested with trypsin at 37°C for 3 h then mixed with formic acid (0.1% final concentration) and analyzed by OrbiTrap XL mass spectrometry. Peptides were identified using PEAKS Studio software (Bioinformatics Solutions, Inc.) and mapped to either an E. coli or Thermococcus kodakarensis protein database (GenBank accession number: NC_006624) (Fig. S1).

### Cloning, expression, purification, and characterization of TK0353.

An E. coli codon-optimized version of TK0353 (NCBI Protein accession number BAD84542.1) was synthetically constructed and cloned into pAII17 vector (31) containing ampicillin resistance via the NdeI and BamHI sites (Genscript). The resulting expression vector containing TK0353 was used to transform T7 Express competent cells (New England Biolabs).

For TK0353 expression, cells were grown in 2L LB medium supplemented with 100 μg/mL ampicillin at 37°C until an optical density at 600 nm (OD_600_) of ∼0.4 was reached. TK0353 expression was then induced with 0.4 mM (final concentration) IPTG. Growth was continued for 3 h at 37°C, and the cells were harvested by centrifugation. The cell pellet was suspended in 20 mM Tris-HCl pH 7.5, 300 mM NaCl and lysed using a cell disruptor (Constant Systems LTD, Northants, UK). The cell lysate was heated to 80°C for 20 min in a water bath and the lysate was clarified by centrifugation.

A five-step purification scheme was used to purify TK0353. The clarified supernatant was loaded onto a HiPrep™ 16/10 DEAE column (GE Lifesciences, Pittsburgh, PA) to remove DNA contamination. The flowthrough was collected, diluted to 20 mM Tris-HCl, pH 7.5, 50 mM NaCl and loaded onto a HiPrep 16/10 Heparin column (GE Lifesciences, Pittsburgh, PA). Bound proteins were eluted using a 50 to 1000 mM NaCl gradient. Fractions containing TK0353 were identified by SDS-PAGE analysis and pooled. This pool was diluted to 20 mM Tris-HCl, pH 7.5, 50 mM NaCl and loaded onto a HiPrep 16/10 Source Q column. Bound proteins were eluted using a Buffer A gradient from 50 to 1000 mM NaCl and the active enzyme was pooled, diluted to 20 mM Tris-HCl, pH 7.5, 50 mM NaCl, and loaded onto a 5 mL HiTrap Heparin column (GE Lifesciences, Pittsburgh, PA). Proteins were eluted using a 50 to 1000 mM NaCl gradient and loaded onto a HiPrep 16/60 Sephacryl S-200 HR sizing column. Peak fractions Fractions containing TK0353 were identified by SDS-PAGE analysis, pooled, and loaded onto a 5 mL HiTrap Heparin column (GE Lifesciences, Pittsburgh, PA). Proteins were eluted using a 50 to 1000 mM NaCl gradient and were dialyzed into 10 mM Tris-HCl, 100 mM KCl, 1 mM DTT, 0.1 mM EDTA, and 50% glycerol, pH 7.4 and protein concentration was quantified using a Qubit fluorometer (Thermo Fisher Scientific, Waltham, MA).

Assays were performed to further characterize the substrate specificity, catalytic mechanism, and metal ion dependence of TK0350. dsDNA templates containing dU:G or THF:G were prepared as described above (Table 1). Reactions (20 μL) reaction mixtures containing 10 nM each dsDNA substrate was incubated with 50 nM purified TK0353 in 1× sodium acetate buffer (10 mM NaOAc, 20 mM NaCl pH 5.5) for 30 min at 65°C. All samples were mixed 10 μL of 50 mM EDTA and diluted 3-fold with ddH_2_O followed by CE analysis as described above.

### Cloning, expression, purification, and characterization of T. kodakarensis Nob1.

The T. kodakarensis Nob1 gene (TK0337) was synthesized by Genscript and cloned into a pET29a(+) plasmid in frame with a C-terminal hexahistidine (His_6_)-tag. The construct was expressed in NiCo (DE3) Competent E. coli cells (New England Biolabs) by growth at 37°C to an optical density at 600 nm (OD_600_) of ∼0.6 before induction with 0.2 mM IPTG for 3 h. The cell pellet was resuspended in breakage buffer (20 mM Tris pH 7.5, 300 mM NaCl) and lysed by two passes through a microfluidizer, and then incubated at 80°C in a water bath for 30 min to inactivate the E. coli proteins. The lysate was then clarified by centrifugation at 10,000 rpm for 15 min at 4°C. All subsequent steps were also carried out at 4°C. The clarified lysate was loaded onto a DEAE column equilibrated with 20 mM Tris-HCl, pH 7.5, 300 mM NaCl to remove DNA contamination. Nob1 was eluted in the wash and flowthrough. The combined wash and flow through from the DEAE were loaded onto a nickel column equilibrated with 20 mM Tris-HCl, pH 7.5, 300 mM NaCl. Nob1 was eluted with a linear gradient of 0 to 500 mM imidazole. Fractions containing Nob1 were determined by SDS-PAGE analysis and pooled. The purified Nob1 enzyme was concentrated using an Amicon Ultra-15 Centrifugal Filter (MWCO 5 kDa, Millipore Sigma) and stored at –20°C in 10 mM Tris-HCl pH 7.4, 100 mM KCl, 1 mM DTT, 0.1 mM EDTA, 50% glycerol.

Purified Nob1 enzyme (1 μM final concentration) was mixed with either 50-mer ssDNA, 50-mer dsDNA, protected 39-mer dsDNA, 50-mer ssRNA, and 50-mer dsRNA substrate (100 nM final concentration) in 1× ThermoPol Buffer and incubated for 20 min at 55°C. The reactions were quenched with 50 mM EDTA and 0.1% Triton X-100. The reaction products were analyzed by capillary gel electrophoresis as described previously (17).

### Data availability.

RNA-seq data for the *Thermococcus kodakarensis* fosmid library in *E. coli* (Plate NEB260) can be accessed at NCBI GEO using accession number GSE182067.
