# Supplementary material for: Capillary Electrophoresis-Based Functional Genomics Screening to Discover Novel Archaeal DNA Modifying Enzymes
Source: Appl Environ Microbiol. 2022 Jan 25;88(2):e02137-21. doi: 10.1128/AEM.02137-21 (PMC8788744; doi:10.1128/AEM.02137-21)
Supplement: Supplemental file 1 — Table S1, Fig. S1 to S4. Download AEM.02137-21-s0001.pdf, PDF file, 0.9 MB [file aem.02137-21-s0001.pdf]

Capillary electrophoresis-based functional genomics screening to discover novel archaeal DNA modifying enzymes

Kelly M. Zatopek[1], Samantha Fossa[1], Katharina Bilotti[1], Paul J. Caffrey[1], Léa Chuzel[1], Alexandra M. Gehring[1], Gregory J. S. Lohman[1], Christopher H. Taron[1], Andrew F. Gardner[1,*].

[1]New England Biolabs, Inc., 240 County Road, Ipswich, MA 01938

* To whom correspondence should be addressed. Tel: 978-380-7262; Fax: 978-921-1350; Email: gardner@neb.com

SUPPLEMENTARY DATA

Supplementary Table 1. Mass spectrometry analysis of proteins contained in peak AP lyase activity fractions during column chromatography.

Description	Coverage (%)	# Peptides	# Unique	Avg. Mass
gb ACT45802.1 chaperonin GroEL [Escherichia coli BL21(DE3)]	65	28	28	57329
gb ACT42985.1 acyl carrier protein [Escherichia coli BL21(DE3)]	79	18	18	8640
gb ACT42077.1 periplasmic chaperone [Escherichia coli BL21(DE3)]	80	16	16	17688
gb ACT43103.1 global DNA-binding transcriptional dual regulator H-NS [Escherichia coli BL21(DE3)]	67	14	13	15540
gb ACT44365.1 carbon storage regulator [Escherichia coli BL21(DE3)]	90	13	13	6856
gb ACT45651.1 50S ribosomal protein L11 [Escherichia coli BL21(DE3)]	33	13	13	14875
gb ACT42116.1 inhibitor of vertebrate C-lysozyme [Escherichia coli BL21(DE3)]	71	11	11	16872
gb ACT42321.1 hypothetical protein ECD_00422 [Escherichia coli BL21(DE3)]	96	10	10	12015
gb ACT44292.1 pyruvate formate lyase subunit [Escherichia coli BL21(DE3)]	75	10	10	14284
gb ACT46057.1 DNA-binding response regulator in two-component regulatory system with ArcB or CpxA [Escherichia coli BL21(DE3)]	37	10	10	27292
gb ACT44941.1 peptide deformylase [Escherichia coli BL21(DE3)]	62	10	10	19328
gb ACT44220.1 inositol-5-monophosphate dehydrogenase [Escherichia coli BL21(DE3)]	28	9	9	52022
gb ACT44959.1 50S ribosomal protein L18 [Escherichia coli BL21(DE3)]	73	9	9	12770
gb ACT43504.1 murein lipoprotein [Escherichia coli BL21(DE3)]	49	9	9	8323
gb ACT45548.1 glutamine synthetase [Escherichia coli BL21(DE3)]	24	9	9	51904
gb ACT44964.1 50S ribosomal protein L24 [Escherichia coli BL21(DE3)]	48	9	9	11316
gb ACT44265.1 regulatory protein P-II for glutamine synthetase [Escherichia coli BL21(DE3)]	52	8	8	12425
gb ACT45654.1 50S ribosomal protein L7/L12 [Escherichia coli BL21(DE3)]	54	8	8	12295
gb ACT44739.1 30S ribosomal protein S21 [Escherichia coli BL21(DE3)]	37	8	8	8500
gb ACT41937.1 dihydrodipicolinate reductase [Escherichia coli BL21(DE3)]	37	7	7	28744

gb ACT41916.1 molecular chaperone DnaK [Escherichia coli BL21(DE3)]	15	7	7	69115
gb ACT43911.1 endonuclease IV [Escherichia coli BL21(DE3)]	28	7	7	31392
gb ACT42856.1 outer membrane protein A (3a;II*;G;d) [Escherichia coli BL21(DE3)]	24	7	7	37201
gb ACT43482.1 hypothetical protein ECD_01624 [Escherichia coli BL21(DE3)]	74	7	7	12879
gb ACT45653.1 50S ribosomal protein L10 [Escherichia coli BL21(DE3)]	48	7	7	17712
gb ACT45648.1 protein chain elongation factor EF-Tu (duplicate of tufA) [Escherichia coli BL21(DE3)]	23	7	7	43314
gb ACT44994.1 protein chain elongation factor EF-Tu (duplicate of tufB) [Escherichia coli BL21(DE3)]	23	7	7	43284
gb ACT42034.1 3-methyl-2-oxobutanoate hydroxymethyltransferase [Escherichia coli BL21(DE3)]	48	7	7	28121
gb ACT42291.1 HU DNA-binding transcriptional regulator beta subunit [Escherichia coli BL21(DE3)]	42	7	7	9226
gb ACT45617.1 transcriptional repressor protein MetJ [Escherichia coli BL21(DE3)]	83	7	7	12141
gb ACT42230.1 hypothetical protein ECD_00331 [Escherichia coli BL21(DE3)]	58	7	7	11667
gb ACT42650.1 molybdopterin biosynthesis protein B [Escherichia coli BL21(DE3)]	47	6	6	18612
gb ACT44969.1 30S ribosomal protein S3 [Escherichia coli BL21(DE3)]	23	6	6	25983
gb ACT44878.1 isoprenoid biosynthesis protein with amidotransferase-like domain [Escherichia coli BL21(DE3)]	43	6	6	22982
gb ACT45606.1 hypothetical protein ECD_03813 [Escherichia coli BL21(DE3)]	64	6	6	9635
gb ACT45891.1 hypothetical protein ECD_04102 [Escherichia coli BL21(DE3)]	54	6	6	21359
gb ACT45652.1 50S ribosomal protein L1 [Escherichia coli BL21(DE3)]	23	5	5	24730
gb ACT44949.1 50S ribosomal protein L17 [Escherichia coli BL21(DE3)]	40	5	5	14365
gb ACT44967.1 50S ribosomal protein L29 [Escherichia coli BL21(DE3)]	87	5	5	7273
gb ACT43537.1 integration host factor subunit alpha [Escherichia coli BL21(DE3)]	43	5	5	11354
gb ACT45415.1 F0F1 ATP synthase subunit B [Escherichia coli BL21(DE3)]	35	5	5	17264
gb ACT43761.1 hypothetical protein ECD_01910 [Escherichia coli BL21(DE3)]	19	5	5	15132
gb ACT45856.1 30S ribosomal protein S6 [Escherichia coli BL21(DE3)]	24	4	4	15173
gb ACT42068.1 30S ribosomal protein S2 [Escherichia coli BL21(DE3)]	14	4	4	26744
gb ACT43563.1 DNA-binding transcriptional activator [Escherichia coli BL21(DE3)]	60	4	4	12021
gb ACT42016.1 dihydrolipoamide dehydrogenase [Escherichia coli BL21(DE3)]	16	4	4	50689
gb ACT45614.1 50S ribosomal subunit protein L31 [Escherichia coli BL21(DE3)]	50	4	4	7871
gb ACT44960.1 50S ribosomal protein L6 [Escherichia coli BL21(DE3)]	27	4	4	18904
gb ACT45045.1 shikimate kinase I [Escherichia coli BL21(DE3)]	34	4	4	19538
gb ACT43543.1 translation initiation factor IF-3 [Escherichia coli BL21(DE3)]	40	4	4	16637
gb ACT42490.1 hypothetical protein ECD_00612 [Escherichia coli BL21(DE3)]	30	4	4	18797
gb ACT44344.1 DNA binding protein nucleoid-associated [Escherichia coli BL21(DE3)]	49	4	3	15348
gb ACT41911.1 molybdenum cofactor biosynthesis protein [Escherichia coli BL21(DE3)]	27	4	4	21164

gb ACT45544.1 hypothetical protein ECD_03751 [Escherichia coli BL21(DE3)]	22	4	4	19059
gb ACT44838.1 ribosome-binding factor A [Escherichia coli BL21(DE3)]	36	4	4	15154
gb ACT42517.1 flavodoxin 1 [Escherichia coli BL21(DE3)]	20	3	3	19737
gb ACT44553.1 glycine cleavage system protein H [Escherichia coli BL21(DE3)]	33	3	3	13811
gb ACT45669.1 HU DNA-binding transcriptional regulator alpha subunit [Escherichia coli BL21(DE3)]	38	3	3	9535
gb ACT44305.1 cold shock protein associated with 30S ribosomal subunit [Escherichia coli BL21(DE3)]	22	3	3	12785
gb ACT43689.1 hypothetical protein ECD_01835 [Escherichia coli BL21(DE3)]	31	3	3	26437
gb ACT42273.1 nucleotide-binding protein [Escherichia coli BL21(DE3)]	24	3	3	18344
gb ACT42811.1 integration host factor subunit beta [Escherichia coli BL21(DE3)]	28	3	3	10651
gb ACT44951.1 30S ribosomal protein S4 [Escherichia coli BL21(DE3)]	12	3	3	23469
gb ACT44963.1 50S ribosomal protein L5 [Escherichia coli BL21(DE3)]	18	3	3	20302
**hypothetical protein TK0353 [Thermococcus kodakarensis KOD1]**	**18**	**3**	**3**	**19611**
gb ACT45769.1 hypothetical protein ECD_03979 [Escherichia coli BL21(DE3)]	30	3	3	12345

```
  1 MYSVKKSKSG YIFDKPRERI AFMFLKDGTY FMYHDGRILC YSLKPVDVSR EELEEFERTG EPPELIKRVK AGKYPENCVV
 81 KELPPIDKGL AQLNPNRKCV IIFTGFQDTV IDYVECNGET LAVARLIDEP GKVCRFAGKG NYKVAAVKLK RNEPCLTREE
                                                    C                                C
161 FLKKVEECRK
```

Supplementary Figure 1. Mapping TK0353 trypsin digest peptide by mass spectrometry. Peptides detected by mass spectrometry (highlighted in grey with blue underlines) were mapped to the TK0353 amino acid sequence. Carbaminomethylation sites are highlighted with an orange "C".

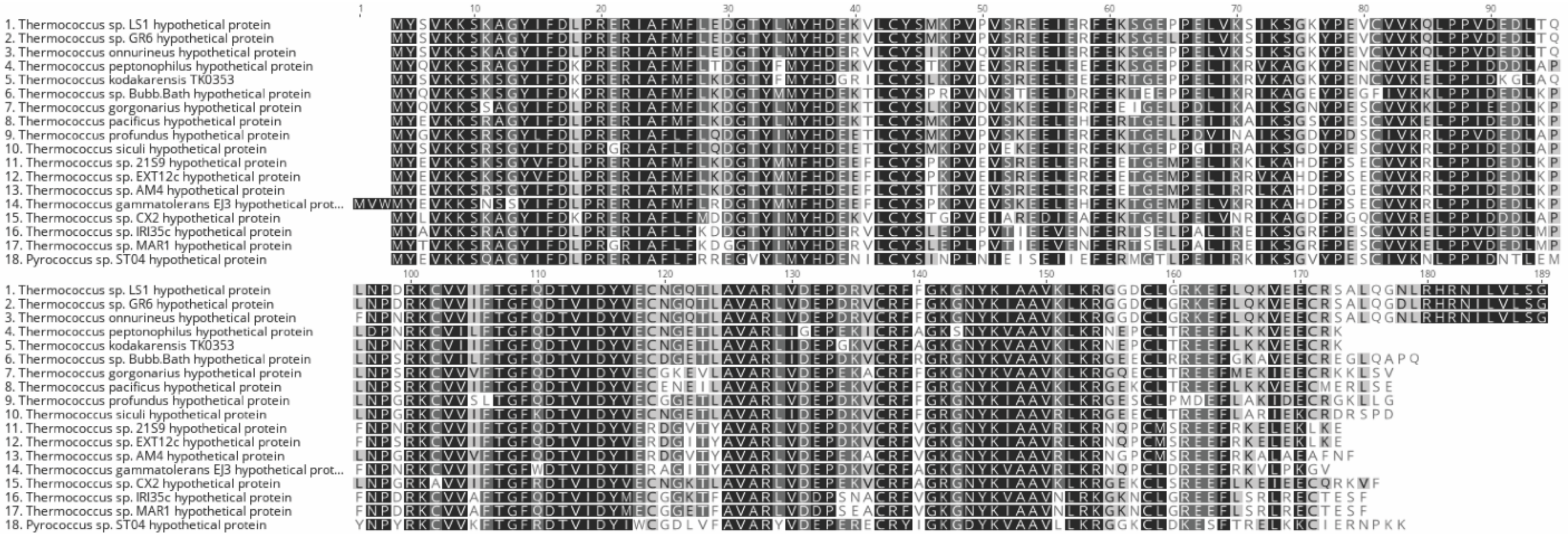

Supplementary Figure 2. TK0353 alignment. TK0353 homologs were aligned using Clustal Omega software.

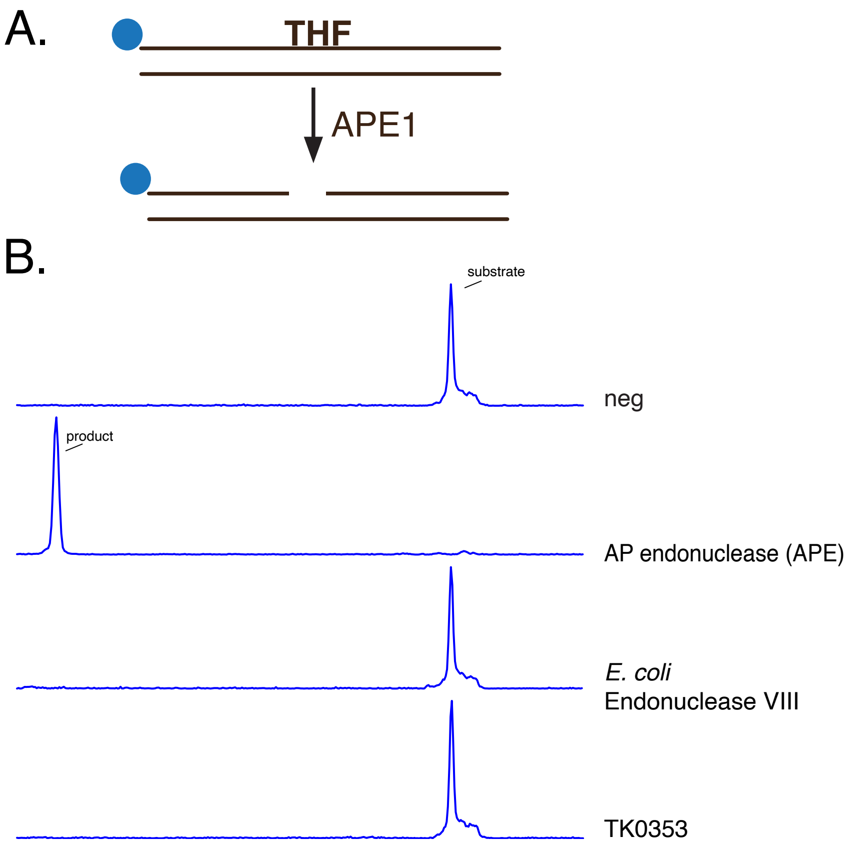

Supplementary Figure 3. TK0353 lacks AP endonuclease activity. (A) A 5'-FAM DNA substrate containing a tetrahydrofuran (THF) AP analog was incubated with no enzyme (neg), AP endonuclease or AP lyases *E. coli* Endonuclease VIII or TK0353 and resolved by capillary electrophoresis. Only AP endonucleases cleave DNA containing a THF site.

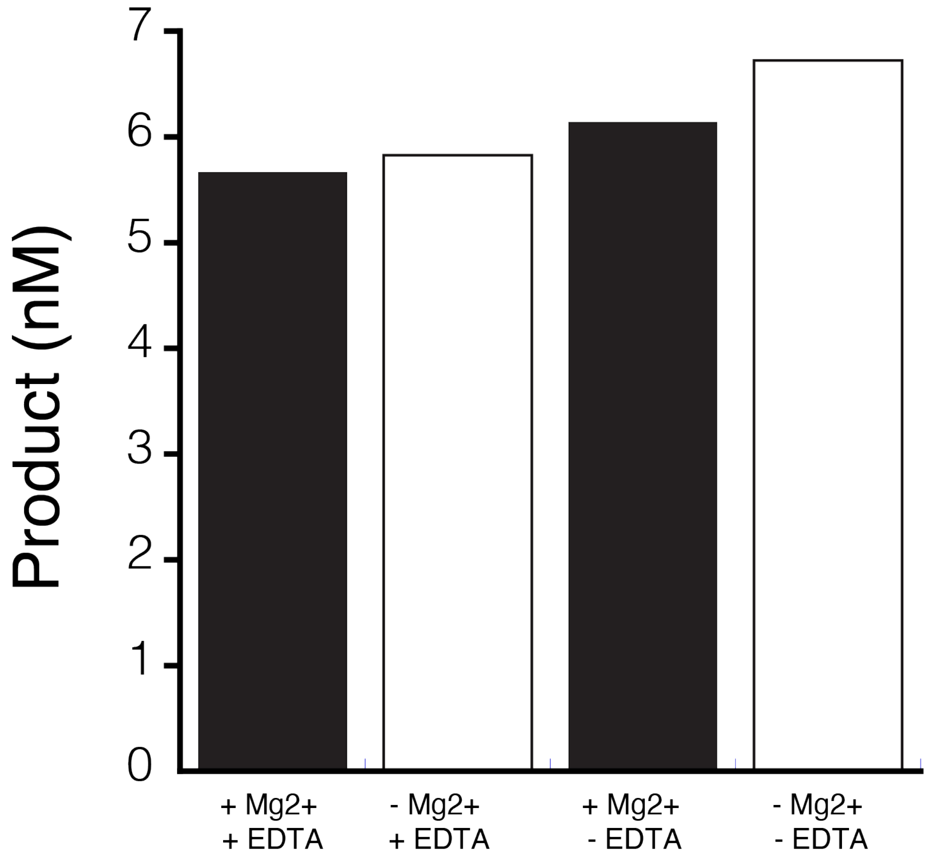

Supplementary Figure 4. TK0353 activity is metal independent. TK0353 activity was monitored as described in Materials and Methods. Reactions contained 0 (- Mg2+) or 2 mM $MgSO_4$ (+ Mg2+) supplemented with 0 (-EDTA) or 50 mM EDTA (+EDTA).
